# Putative filariosis outbreak in white and black rhinoceros at Meru National Park in Kenya

**DOI:** 10.1186/1756-3305-5-206

**Published:** 2012-09-19

**Authors:** Matthew Mutinda, Moses Otiende, Francis Gakuya, Linus Kariuki, Vincent Obanda, David Ndeere, Ephantus Ndambiri, Edward Kariuki, Isaac Lekolool, Ramón C Soriguer, Luca Rossi, Samer Alasaad

**Affiliations:** 1 Department of Veterinary and Capture Services, Kenya Wildlife Service, Nairobi, Kenya; 2 Estación Biológica de Doñana, Consejo Superior de Investigaciones Científicas (CSIC), Avda. Américo Vespucio s/n 41092, Sevilla, Spain; 3 Dipartimento di Produzioni Animali, Epidemiologia ed Ecologia, Università degli Studi di Torino, Via Leonardo da Vinci 44, Grugliasco, I-10095, Italy; 4 Institute of Evolutionary Biology and Environmental Studies (IEU), University of Zürich, Winterthurerstrasse 190, Zürich, 8057, Switzerland

**Keywords:** Filariosis, *Stephanofilaria dinniki*, *Diceros bicornis*, *Ceratotherium simum*, Treatment, Threatened species

## Abstract

**Background:**

Habitat and food supply loss and disruption, together with man’s pursuit of the animal’s unique horn pose significant threats to the charismatic rhinoceros. Filarial worms have been thought to cause cutaneous lesions in black rhinoceros (*Diceros bicornis*) in Kenya and South Africa, but never in white rhinoceros (*Ceratotherium simum*) in the wild, despite the fact that the two species live often in close proximity. *Stephanofilaria dinniki* has been implicated in the past as the causal agents for such lesions.

**Findings:**

In this paper we report a putative filariosis outbreak in both black and white rhinos at Meru National Park in Kenya. Four black and five white rhinos were affected by various degrees of filarioid-like lesions, while apparently all sympatric wild and domestic animals were filarial worm-free. Affected rhinos were captured and successfully treated. Comparison between the epidemiological aspects of white and black rhinoceros filariosis, and the possible relations between this outbreak and annual seasons, the presence of oxpeckers and other host species are discussed.

**Conclusions:**

Our study highlights (i) that filarial infection is not restricted to black rhinos, but it affects both rhinoceros species, and (ii) the importance of the earlier detection and immediate treatment (capture-treat and release) of filarioid infections, which is of pivotal interest for wildlife conservation, and especially the endangered and isolated white and black rhinoceros populations.

## Findings

The world rhinoceroses’ population has been reduced by more than 90% only in the past 30 years. From the 30 known rhinoceros species once inhabiting our planet, only five threatened species persist today. Only the black (*Diceros bicornis*) and white rhinoceros (*Ceratotherium simum*) still roam in Africa. The dreadful plight of this charismatic species is due to habitat and food supply loss and disruption, and mainly because of man’s pursuit of the animal’s unique horn, which poses the single most dangerous threat [1-3].

The causal agent of the frequent occurrence of ulcerative wounds behind the shoulder of black rhinoceros in Kenya and South Africa has been the subject of speculation for many years. The seasonal appearance of the lesions led to the belief that they were associated with secondary sex skin glands, which became active during the breeding season [4,5]. *Stephanofilaria dinniki* has been implicated in the past as the causal agent for such lesions [6,7]. To the best of our knowledge, in all reports only black rhinos have been shown to be affected and not the white rhino species [8], despite the fact that the two species live often in close proximity [6]. Even so, filarial lesions in white rhinos at Meru National Park (Kenya) have been reported in captivity [9].

The aims of the present study are: (i) to describe for the first time filariosis simultaneous outbreaks in white and black rhinos from wild populations, (ii) to report the efficacy of filarid infection treatment in this wild animal.

### Meru National Park and Rhino Sanctuary, Kenya

This National Park is situated in northern Kenya, covers an area of 870 square kilometres. It has abundant rainfall, 635–762 mm in the west of the park and 305–356 mm in the east. The rainfall results in tall grass and lush swamps, which make it difficult to spot wild animals. It has a wide range of wild animals like elephant, hippopotamus, lion, leopard, cheetah, black rhinoceros and some rare antelopes, with incursions from cattle, camel, goats and sheep. The Meru National Park is a home for 22 black rhinos and 48 white rhinos. During the dry season there are constant incursions of livestock that include goats, camels and sheep in the park area.

### Case report

**Figure 1:**
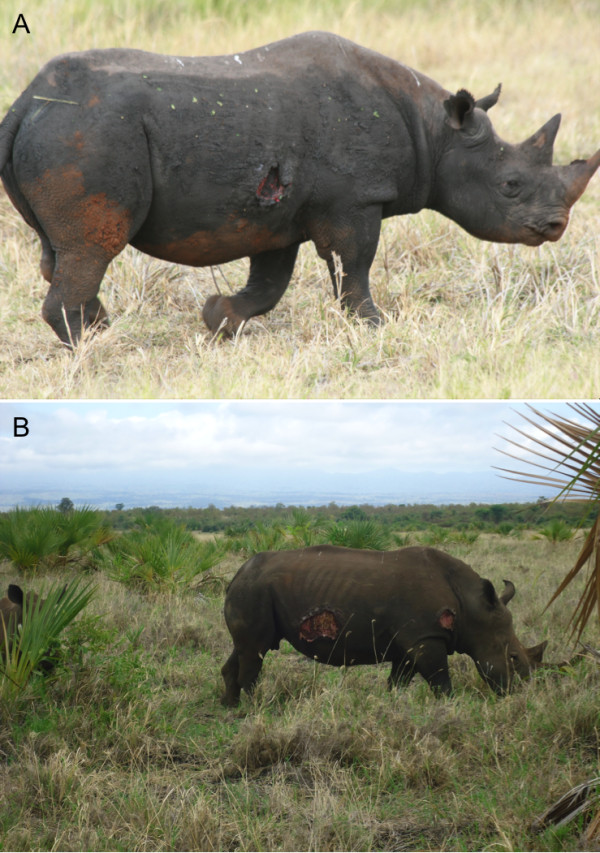
Extensive filarial like lesion in (A) a black rhino, Toyo, and (B) a white rhino, Stella.

In May 2011 during our routine monitoring of the health status of wild and domestic animal populations in Meru National Park, we identified five white and four black (Figure 1) rhinos with filariosis-like lesions, while apparently all other sympatric wild and domestic animals were filarioid-free. The affected white rhinos were 2 males and 3 females, and the infected black rhinos were 3 males and 1 female. The affected rhinos were between 3.5 and 27 years old (age estimation was based on known morphometric criteria; [10]).

The spatial distribution of the infected white and black rhinos within Meru National Park was scattered and isolated points with an average distance of 3 km ranging between 2 and 4 km (No map or GPS references were giving in this paper for security reasons, and to protect these rhinoceroses of being poached).

### Macroscopic examination of the filariosis-like lesions and parasite collection

**Figure 2:**
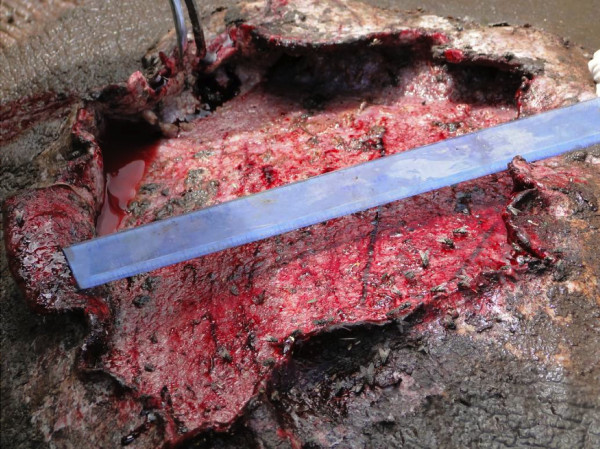
A white rhino, Rosie, wound at time of treatment showing macroscopic appearance and size of filarial like lesion.

Attempts were made to collect worms surgically from the subcutis of the immobilized rhino, and small pieces of the affected skin were collected for histopathological examination. The lesions were characterized by erosive ulcerations and crust formation. The affected skin area appeared to peel off towards the healthy side with dry and crusty edges falling off leaving a massive reddish area with neither smell nor maggots. Wounds apparently healed up well but left a big scar. The central area of the lesion was depressed and the peripheral edge of the lesion rose above the normal edge of the skin. There was a difference in thickness between the normal area of the skin and the lesion area of about 2–3 cm [11], (Figure 2).

The wounds were quite big; the mean size was 23 ± 8 cm in diameter in the white rhinos, while the black rhinos had much smaller wounds of 15 ± 5 cm. The location of the wounds was varied; white rhinos had massive wounds on the rump, behind the shoulder and the axillary regions, while the black rhinos had wounds on the ribs and behind the shoulder. Red-billed oxpeckers were observed in both rhino species.

### Filariae-infected rhinos capture and treatment

Infected rhinos were darted with Dan-inject® dart gun from a helicopter. The dart contained 5 mg Etorphine Hydrochloride (M99®) and 30 mg Xylazine (ILIUM, Troy Laboratories, Australia.) for adult black rhinos and 6 mg Etorphine Hydrochloride and 50 mg Xylazine for adult white rhinos. The animals went down in approximately 6 minutes. After getting down, anaesthetic plan was improved by injecting 5 mg of Nalorphine into an ear vein. In case of the presence of accompanying calves, they were driven away by the help of a vehicle. The animals were then placed on sternal or lateral recumbency to be sampled and treated.

**Figure 3:**
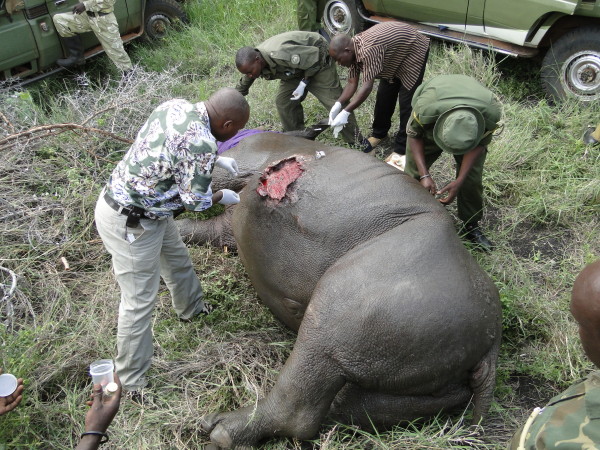
Photo showing treatment and sampling of an immobilized white rhino.

Each rhino was treated with 15,000 mg intramuscularly, long acting Amoxicillin Trihydrate (Betamox®), Ivermectin 200 mg (dosage of 1 ml per 50 kg body weight) and local wound treatment, cutting dead tissue debridement followed by spraying with povidone iodine and fly repellent in some wounds (Figure 3).

Animals were revived by the injection of 18 mg Diprenorphine (M5050®) and 6 mgs Atipamezole Hydrochloride (ANTISEDAN®) into an ear vein. The white rhinos were also injected with 25 mg Naltrexone Hydrochloride (Naltrexone; Kyron Laboratories) intramuscularly to prevent re-narcotisation. All rhinos got up in approximately three minutes.

## Results and Discussion

In the present study we report for the first time a putative filarid outbreak in both black and white rhinos from Meru National Park in Kenya. We failed to collect worms surgically from the subcutis of the immobilized rhinos, and the histopathological examination was negative for the collected samples. Only *Stephanofilaria dinniki* has been implicated in the past as the causal agent for such filarioid-lesions, and hence we expect this parasite species to be the causative agent of the outbreak of this disease.

The life cycle of *S dinniki* parasite is unknown. Species of the order to which it belongs require blood-sucking arthropods to complete the cycle [12]. Members of the phylum Arthropoda found associated with rhino are mosquitos, flies and ticks, and hence they can be considered in the transmission of this parasite. Oxpeckers have been seen to peck at the lesions but they are probably not involved in the life cycle but may be attracted by ticks and loose skin. Further studies are needed to understand the life cycle and the vectors of this parasite.

When living conditions deteriorate for hosts, during periods of overcrowding or food shortage, animal become stressed. This stress has often been linked to epidemics, which are attributed to the immuno-compromised status of the stressed hosts [13-15]. In addition, the prevailing environmental conditions could have contributed to lesions and possible filariosis outbreak in both rhino species in Meru National Park. The Rhino Sanctuary at the time of the outbreak was wet, bushy and thick, compounded by dense under growth, while the open areas were most likely swampy patches. Kock & Kock [16] reported that poor body condition and heavy rainfall were thought to predispose to development of recrudescence of lesions.

**Figure 4:**
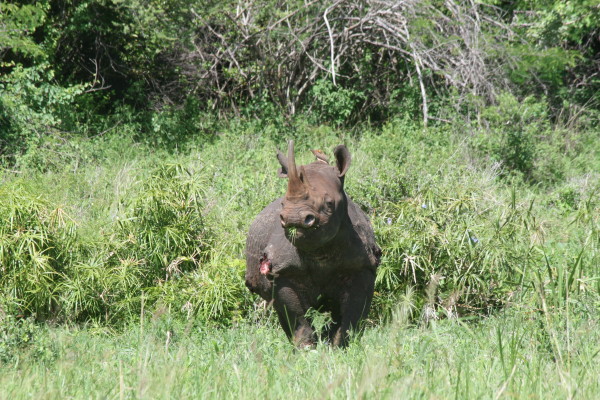
A black rhino, Doreen, showing signs of filarid-like lesions, together with the presence of an oxpecker.

The relationship between oxpeckers and the large mammalian species in Africa is, controversially, usually classified as “cleaning symbiosis” as it is thought to be equally beneficial to both species [17,18]. Keet *et al*. [19] reported that red-billed (*Buphagus erythrorynchus*) oxpeckers could play an important role in the pathogenesis of filarioid epidemiology and lesions. The carnivorous behaviour of these birds, ingesting the blood containing embryonated eggs, could reduce the likelihood of parasite spread, but at the same time they feed on the superficial necrotic skin causing the development of larger ulcers. In the white and black rhinos we observed with lesions we also observed the presence of yellow- and red-billed oxpeckers, which could possibly play an important epidemiological role in filarioid control (Figure 4).

**Figure 5:**
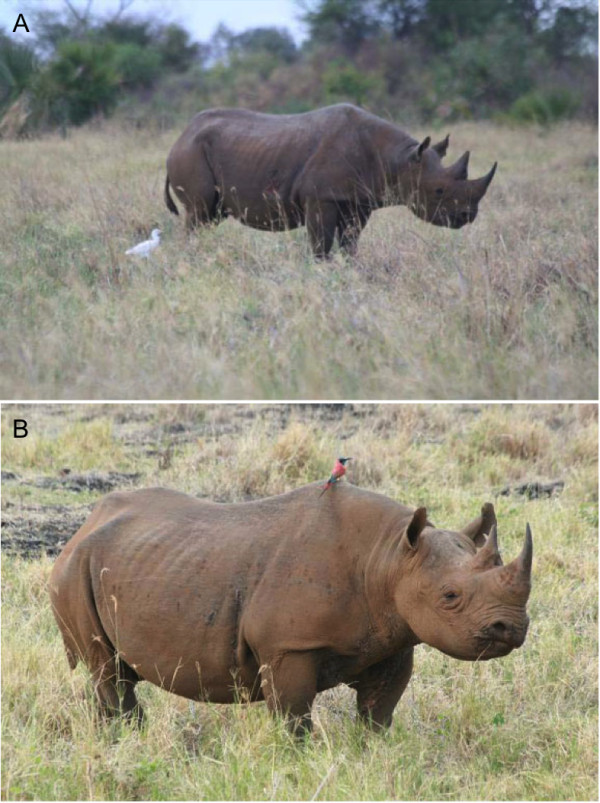
A black rhino, Kelele, (A) before and (B) after treatment.

The treated rhinos recovered completely within three months, and no new cases presented in the last monitoring in May 2012. Rhinoceros recovery was evaluated by the reductions in the mean number and surface area of lesions (Figure 5). This shows that the disease can be controlled in an ecosystem via therapeutic treatment of positive cases “capture-treat and release”, which is in concordance with other studies showing the high efficacy of ivermectin against other filarioid infections, but it has not previously been tested in rhinoceros species [20-22].

One explanation of the high frequency of filarioid outbreaks in wildlife, during the last decades, could be the intrusion of humans and their livestock into wildlife habitat ranges, resulting in great changes in the interface between wildlife and people/livestock, and wildlife habitat losses, and hence the interaction between human/livestock and wildlife is changing from often sporadic and fragile to more permanent and substantial, providing significant opportunities for parasite transmission [23]. The permanent loss of wildlife genetic diversity could have contributed negatively, making wild animals more susceptible to parasite infection [24,25].

The presence of parasites in any ecosystem generates complex parasite webs within the system, and it is through these webs that zoonotic parasites move from one host to other [26,27]. Hence, more studies are needed to understand the filarioid-navigating web including potential molecular analyses [28,29], and the range of underlying causal factors of its unexplicable emergence and re-emergence [24,30], which poses a substantial threat to the conservation of the global biodiversity [23].

### Ethics

The Committee of the Department of Veterinary and Capture Services of the Kenya Wildlife Service (KWS) approved the study including animal capturing and treatment protocols. KWS guidelines on Wildlife Veterinary Practice-2006 were followed. All KWS veterinaries were guided by the Veterinary Surgeons Act Cap 366 Laws of Kenya that regulates veterinary practices in Kenya.

## Competing interests

The authors declare that they have no competing interests.

## Authors’ contributions

MM, MO, FG, LK and VO conceived and designed the experiments. MM, MO, FG, LK, VO, DN, EN, EK, IL, RCS, LR and SA performed the fieldwork experiments. Manuscript was analysed, discussed and written by all co-authors. All authors read and approved the final manuscript.
